# Monthly Reports of Deaths in Buffalo for July 1851

**Published:** 1851-09

**Authors:** W. L. G. Smith

**Affiliations:** Clerk


*Monthly Report of Deaths in Buffalo for July* 1851.

BOARD OF HEALTH, *Buffalo*, August, 1851.

Number of deaths reported to the Board of Health for the month of July last:—Males, 56; females, 31 — total, 87. Age — Adults, 21; children, 66. Diseases — Consumption, 8; scarlet fever, 1; small pox, 1; typhus fever, 2; pneumonia, 1; dysentery, 6; cholera infantum, 3; apoplexy, 1; jaundice, 1; summer complaint, 15; unknown, 12; puerperal fever, 1; cholera morbus, 1; congenital syphilis, 1; convulsions, 8; dentition, 2; poison, 1; disease of the heart, 1; inflammation of the bowels, 2; inflammation on the brain, 2; drowned, 2; bilious fever, 1; marasmus, 2; puerperal mania, 1; hydrocephalus, 3; old age, 2; still-born, 1; whooping cough, 1; dropsy, 1.

By order of the Board,

W. L. G. SMITH, Clerk.

